# Visuo-tactile integration in autism: atypical temporal binding may underlie greater reliance on proprioceptive information

**DOI:** 10.1186/s13229-015-0045-9

**Published:** 2015-09-14

**Authors:** Katie Greenfield, Danielle Ropar, Alastair D. Smith, Mark Carey, Roger Newport

**Affiliations:** School of Psychology, The University of Nottingham, University Park, Nottingham, NG7 2RD UK

**Keywords:** Amodal, Autism spectrum disorders, Multisensory integration, Proprioception, Sensory processing, Temporal binding window

## Abstract

**Background:**

Evidence indicates that social functioning deficits and sensory sensitivities in autism spectrum disorder (ASD) are related to atypical sensory integration. The exact mechanisms underlying these integration difficulties are unknown; however, two leading accounts are (1) an over-reliance on proprioception and (2) atypical visuo-tactile temporal binding. We directly tested these theories by selectively manipulating proprioceptive alignment and visuo-tactile synchrony to assess the extent that these impact upon body ownership.

**Methods:**

Children with ASD and typically developing controls placed their hand into a multisensory illusion apparatus, which presented two, identical live video images of their own hand in the same plane as their actual hand. One virtual hand was aligned proprioceptively with the actual hand (the veridical hand), and the other was displaced to the left or right. While a brushstroke was applied to the participants’ actual (hidden) hand, they observed the two virtual images of their hand also being stroked and were asked to identify their real hand. During brushing, one of three different temporal delays was applied to either the displaced hand or the veridical hand. Thus, only one virtual hand had synchronous visuo-tactile inputs.

**Results:**

Results showed that visuo-tactile synchrony overrides incongruent proprioceptive inputs in typically developing children but not in autistic children. Evidence for both temporally extended visuo-tactile binding and a greater reliance on proprioception are discussed.

**Conclusions:**

This is the first study to provide definitive evidence for temporally extended visuo-tactile binding in ASD. This may result in reduced processing of amodal inputs (i.e. temporal synchrony) over modal-specific information (i.e. proprioception). This would likely lead to failures in appropriately binding information from related events, which would impact upon sensitivity to sensory stimuli, body representation and social processes such as empathy and imitation.

## Background

Hypo- and hypersensitivities to sensory stimuli are prevalent in autism spectrum disorders (ASD) such that they are now a diagnostic criterion in the Diagnostic and Statistical Manual of Mental Disorders (fifth edition) (DSM-5) [1]. Evidence suggests that such sensory disturbances could be due to atypical multisensory integration (MSI): the process of combining sensory input to construct a comprehensible and unified representation of the world [2]. A growing literature indicates atypical MSI in ASD (see [2, 3] for a review), although the majority of research has focused on visuo-auditory integration. Far less is known about the mechanisms underlying atypical visual, tactile and proprioceptive integration in ASD. This is particularly important to establish since the capacity to compare and differentiate between the self and others depends on the normal integration of proprioceptive, somatosensory and visual inputs [4]. This ability, and a sense of body ownership, underlies the development of social behaviours and skills including self-awareness, imitation and empathising [5] which are compromised in ASD [1]. Therefore, atypical integration of these inputs could underlie both sensory and social deficits observed in the disorder, offering an explanatory mechanism that could account for both low-level and high-level components of the ASD behavioural profile. Although the processes underlying this remain elusive, two prominent accounts of atypical multisensory integration in ASD are (1) an over-reliance on proprioception and (2) atypical visuo-tactile temporal binding. We directly tested both of these theories in the present study.

### Over-reliance on proprioception

This account is based on a fundamental bias towards one sensory modality, rather than processing and integrating input from multiple sensory modalities [6]. Such an approach supports the weak central coherence theory [7], a prominent cognitive explanation of ASD, and coheres with numerous findings of superior abilities in low-level perceptual tasks [7]. In particular, a specific bias for (or over-reliance on) proprioception has been implicated in ASD through research in which children learn to control a robotic arm to reach towards a target. Haswell et al. [8] showed that typically developing (TD) children were more likely to integrate visual and proprioceptive feedback to achieve the task while children with ASD placed a greater reliance on proprioception alone. Using a similar task, Marco et al. [9] included trials in which reaching actions were perturbed resulting in movement errors sensed through visual and proprioceptive systems. Results showed that sensitivity to proprioceptive error was significantly larger in children with ASD compared to TD controls while the reverse was true for sensitivity to visual error.

Although additional studies support the idea of an over-reliance on proprioceptive feedback and an under-reliance on visual inputs [10, 11], other investigations (e.g. [12, 13]) have not consistently found support for this theory. Weimer et al. [13], for example, reported that children with ASD performed worse than TD children on tasks in which a lack of visual information necessitated dependence on proprioceptive feedback alone, such as one-leg balancing with eyes closed, which would not be expected if individuals with ASD had a bias towards processing unimodal, proprioceptive inputs.

### Atypical visuo-tactile temporal binding

An alternative leading theory of atypical MSI proposes that sensory temporal binding is atypical in ASD. Evidence for temporally extended binding has been found for both social and non-social visual-auditory integration in ASD (e.g. [14–16], but see [17]). Temporal binding refers to the interval within which two or more sensory inputs can be integrated. For example, even when a visual input and an auditory input do not occur at exactly the same time, these can still be integrated and perceived as one multimodal event. However, if the temporal distance between sensory events is too large, these will be perceived as two separate events. Temporally extended binding would likely lead to inappropriate integration of information from unrelated events which could be underlying the feelings of sensory overload commonly seen in the disorder [18]. However, one key, but as yet unanswered, question is whether the temporal binding of visual and *tactile* information is atypical in people with ASD. This is necessary to assess whether the theory is specific to visuo-auditory integration or if it can account for atypical MSI across multiple sensory modalities.

To our knowledge, only two studies have directly investigated visuo-tactile-proprioceptive processing in ASD [4, 19]. Both have used the rubber hand illusion (RHI) in which temporally congruent seen and felt brushstrokes are applied to a fake hand and a real hand, respectively. In this effect, TD adults are guided by visuo-tactile temporal synchrony, even when this information is incongruent with proprioceptive information, leading to ownership of the fake limb [20]. Paton et al.’s [19] RHI study showed that adults with ASD displayed not only reduced embodiment of the rubber hand but also more accurate localisation estimates of their hidden hand than a control group, which could indicate a bias towards proprioceptive processing. Cascio et al. [4] found a delayed onset of the illusion in children with ASD compared to TD controls, which they suggest may be due to an extended time window for visuo-tactile integration. Though findings from both studies suggest atypical visual-tactile-proprioceptive integration in ASD, the classic RHI paradigm cannot distinguish evidence for an over-reliance on proprioceptive processing over temporally extended visuo-tactile binding as both explanations would predict reduced susceptibility to the illusion. Moreover, in both Paton’s and Cascio’s studies, brushing was conducted in sets of 3-min blocks during which participants were required to keep their hand still and attend to the fake hand throughout. Since atypical attention is common in ASD [21], reduced sustained attention to the fake hand could have contributed to group differences. Additionally, the imagination deficits characterising ASD [1] may play a role in reduced illusion susceptibility. The classic RHI requires an individual to overcome the discrepancies in physical characteristics between the fake and real hand (i.e. texture, shape), which impact on the extent to which the rubber hand is embodied [22]. Such differences may be more salient for individuals with ASD since detail-focused processing is characteristic of this population [7, 23] and thus could also underlie reduced embodiment of the rubber hand. The current study used a unique technique that avoided these inherent limitations of the classic RHI design and allowed us to clearly and separately distinguish evidence for an over-reliance on proprioceptive processing and temporally extended visuo-tactile binding.

## Methods

### Participants

Participants (see Table 1 for participant characteristics) included 31 children with ASD aged 8–15 years (2 female, 1 left-handed), 29 chronological age-matched (CA) controls (8 female, 5 left-handed) and 29 verbal mental-age-matched (MA) controls aged 5–10 years (10 female, 2 left-handed). Individuals with ASD were recruited from autism support groups and a local school in Nottingham. Comparison participants were (*n* = 40) recruited from Summer Scientist Week, a community event held at The University of Nottingham or from the university’s database of local families. The British Picture Vocabulary Scale III [24] was used to assess verbal MA in all groups. MA data was missing for one participant in the CA group. There were no significant differences in verbal mental age between the ASD and MA group or in chronological age between the ASD and CA group. As we tested individuals with ASD who varied in their cognitive abilities, we calculated developmental quotient [25] to give an indication of the range of delay in the group (see Table 1).

**Table 1:** Participant characteristics for chronological age (CA)-matched, verbal mental age (MA)-matched and autism spectrum disorder (ASD) groups

Group (sample size)	Statistic	Age in months	Verbal mental age in months	SAS	SWAN	SCQ	Developmental Quotient
ASD (29)	Mean	151.65	103.17	10	0.77	24.64	69
	SD	23.07	37.37	5.90	0.66	5.2	24.43
	Min	99.72	59.0	0	−0.39	15	38.10
	Max	191.04	189.0	23	2.67	34	134.04
CA-matched (29)	Mean	146.13	150.5	24.71	0.35	Not collected	N/A
	SD	21.35	35.19	6.17	0.75		
	Min	101.0	81.0	13	−1.89		
	Max	184	189.0	40	0.85		
MA-matched (29)	Mean	94.56	100.35	25.71	−0.76	Not collected	N/A
	SD	16.68	27.33	5.71	0.96		
	Min	63.48	64	19	−2.78		
	Max	123.6	172	39	0.78		

*SAS* Social Aptitudes Scale, *SWAN* Strengths and Weaknesses of ADHD Symptoms and Normal Behavior Scale, *SCQ* Social Communication Questionnaire. SWAN scores ranged from −3 to 3; higher scores indicate a higher level of ADHD symptoms

The study was conducted in accordance with the Declaration of Helsinki at the time the data were collected (Version 6, 2008). All parents of participating children and their schools consented to taking part in the study, which was approved by The University of Nottingham, School of Psychology ethics committee.

### Inclusion/exclusion criteria

All children in the ASD group had received a previous diagnosis of autism, autism spectrum disorder or Asperger syndrome by an independent clinician using the ADOS or ADI-R. Confirmation of diagnosis was obtained via a parent/caregiver in a background questionnaire and additionally through parents’ ratings on the Social Communication Questionnaire (SCQ [26]) and the Social Aptitudes Scale (SAS [27]). Two individuals did not return the completed questionnaires; however, as they were recruited from a specialist autism unit requiring a formal diagnosis and statement of special educational needs, it is unlikely they did not have ASD.

Children in all groups were screened for other developmental difficulties (e.g. motor, attention, visual, language delay) via a parental background questionnaire. Additional screening was carried out for attention deficit hyperactivity disorder using the Strengths and Weaknesses of ADHD Symptoms and Normal Behavior Scale (SWAN) questionnaire [28] and for social deficits using the SAS [27]. None of the typically developing children had a diagnosis of ASD or any other learning difficulty, confirmed by parent questionnaire or additional screening measures; therefore, all were included. In the ASD group, one individual had dyspraxia, one had dyslexia, one had ADHD and one was reported to have hypermobile joints.

There were several criteria participants were required to meet to be included in the study. Firstly, all needed to have normal or corrected-to-normal vision. Secondly, all participants took part in practice trials where they needed to demonstrate (1) an ability to keep their hand still and (2) an ability to understand the task questions. Two children from the ASD group were excluded, as they could not keep their hand still to complete the task, leaving 29 ASD children whose results were included in the analyses.

### Procedure

All children completed a task using a MIRAGE-mediated reality device that presents live video images of the hand in real time as if viewing the hand directly; that is, in the same spatial location and from the same visual perspective. Real-time video is acquired and manipulated online using a powerful combination of custom-made hardware and software that can control visual presentation with ms precision.

Depending on their height, participants sat or knelt on a chair to allow them to comfortably view their right hand when they placed it onto the work surface of the MIRAGE. A rectangular horizontal mirror suspended above the work surface reflected a computer screen suspended above the mirror, facing downwards. Live video images of the participant’s moving hand were displayed on the monitor and reflected via the mirror to appear as direct visual feedback of actual movements, giving the participant the impression that they were viewing their own hand. A rectangular black bib was attached across the length of the MIRAGE, on the side that the participant was seated, to obscure the work surface from view. Participants wore a black adjustable sleeve, which covered their right wrist and forearm, ensuring that only the hand was visible when their arm was in the MIRAGE. Firstly, children completed practice trials in which they placed their right hand into the MIRAGE and saw two virtual representations of their hand. These trials were identical to experimental trials described below except that neither hand image showed a visual-tactile delay. These were included to ensure that participants were comfortable with the set-up and understood the task requirements.

**Fig. 1:**
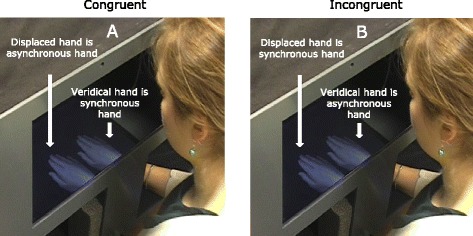
MIRAGE task. The participant placed his/her right hand into the MIRAGE and saw two live video images of their hand. The veridical hand was in the same location as his/her actual hand; the displaced hand was immediately to the left or right of the veridical hand (position of the displaced hand was counterbalanced). In congruent conditions (**a**), the displaced hand had a temporal delay of either 60, 180 or 300 ms applied to it (asynchronous hand); the veridical hand did not (synchronous hand). In incongruent conditions (**b**), the veridical hand had a temporal delay of either 60, 180 or 300 ms applied to it (asynchronous hand); the displaced hand did not (synchronous hand). The arm is here uncovered for illustrative purposes, but it was covered in the experiment so that participants were unable to see the exact relationship between the limb and image

In the experimental trials, we selectively manipulated proprioceptive alignment and visuo-tactile synchrony to explore the extent to which these impact on body ownership. Proprioceptive alignment was manipulated by presenting one hand (the veridical hand) in the same location as the child’s actual hand while a duplicate hand was displaced immediately to the left or right of this (displaced hand; see Fig. 1). Since hand sizes varied between children, the displaced hand was located such that the two hands did not overlap and also that there was a visible <5-mm gap between the hands. That is, the hands were immediately adjacent to each other.

The experimenter brushed the participants’ right index finger with a paintbrush at 1 Hz for 10 s while they saw the brushstrokes on both right hands. After brushing, a yellow shape appeared above one hand image and a different red shape appeared above the other. The images were a circle or a square and their location, colour and shape were counterbalanced for each trial. Participants were reminded to keep their hand still and asked to verbally name the shape they thought was above their real hand. After responses were given, vision of the hand was occluded while the experimenter placed the participant’s hand at the starting point for the next trial. Previous MIRAGE studies employing this supernumary illusion demonstrate that a brushing time of 20 s is sufficient for participants to embody the synchronous hand [29–31]. However, piloting for the current study revealed that individuals distinguished and embodied the synchronous hand after only 10 s of brushing. Additionally, the effect is consistently seen in children at public engagement events when brushing is less than 10 s. Thus, to keep testing time to a minimum, brushing lasted 10 s in all conditions of the current study.

On each trial, visuo-tactile synchrony was manipulated by applying a temporal delay of either 60, 180 or 300 ms to either the veridical or the displaced hand. Thus, the felt brushstrokes were synchronous with the visual brushstrokes on that hand (the synchronous hand) but asynchronous on the other hand. For each condition, therefore, either the veridical hand or the displaced hand was the synchronous hand, while the other hand had a temporal delay applied to it. Delay rates were calculated and monitored online and required no mechanical apparatus. The precise delay was calibrated using software ‘probes’ which can determine the number of milliseconds that have elapsed at any given stage within the programme cycle. The delay is only applied to one of the visual presentations of the hand on each trial. Therefore, even if the real brushstroke is not at a fixed frequency, the seen delayed brushstroke will always follow at the set time after. Based on average child hand sizes, the visual angle subtended from finger to finger would have been in the region of 7.5–10 °, depending on actual hand size. From left thumb to right little finger would have been twice this.

**Fig. 2:**
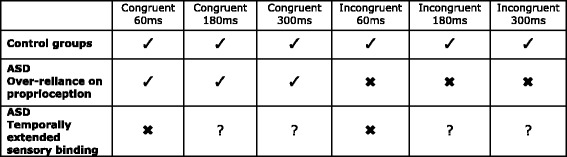
Predictions. Key: *Ticks* = choose synchronous hand significantly above chance. *Question marks* = may choose synchronous hand significantly above chance. *Crosses* = do not choose the synchronous hand significantly above chance. Children in the control groups were predicted to choose the synchronous hand across all conditions, provided they could detect and distinguish it from the asynchronous hand. If children in the ASD group have an over-reliance on proprioception, they should choose the synchronous hand in all the congruent conditions but in none of the incongruent conditions. If children in the ASD group have temporally extended visuo-tactile binding, they should choose the synchronous hand in both congruent and incongruent conditions, but only at longer delay lengths, relative to the control group

In congruent conditions, visuo-tactile inputs were synchronous for the veridical hand (congruent proprioceptive and visuo-tactile input) while the visual touch on the displaced hand was delayed. In incongruent conditions, the visual touch on the veridical hand was delayed; therefore, proprioception and information from visuo-tactile synchrony were incongruent. There were six conditions in total (see Fig. 2; congruent 60-, 180- and 300-ms delay and incongruent 60-, 180- and 300-ms delay). For each condition, the displaced hand was presented once to the left of the veridical hand and once to the right of it. Trials and conditions were presented in a randomised order.

### Hypotheses

Predicted performance for the control and ASD groups is shown in Fig. 2. A recent RHI study showed that, like adults, TD children integrate synchronous visual and tactile inputs to embody a fake hand even when this necessitates overcoming proprioceptive incongruity [32]. There is no ‘real’ and ‘fake’ hand distinction in the current paradigm. However, in similar MIRAGE experiments in which a temporal delay was applied to the asynchronous hand, adults consistently embodied the synchronous hand, even when it was not presented in the location of their actual, unseen hand [29, 30]. Thus, in line with these findings, it was predicted that the TD children would integrate the felt brushstrokes with the visually synchronous brushstrokes and hence choose the synchronous hand in both the congruent and incongruent conditions.

To embody the synchronous hand, children must detect the visual delay applied to the asynchronous hand and discriminate this from the synchronous hand. Temporal-order judgment tasks demonstrate that TD adults can detect visuo-tactile temporal discrepancies as small as 28 ms [33]. Since the current study was a novel task necessitating embodiment of a virtual hand (for which delays of 300 ms have been suggested [34]), piloting was conducted to ascertain the delay lengths applied to the asynchronous hand necessary for it to be discriminated from the synchronous hand. Data from 15 adults showed that, in congruent conditions, most participants chose the synchronous hand and the number doing so increased with delay length (*n* = 9 at 60 ms, *n* = 14 at 180 ms, *n* = 15 at 300 ms). These delay lengths were thus chosen for the current study to compare group performance on conditions requiring differing degrees of sensitivity to visuo-tactile synchrony. It was thus further predicted that TD children would choose the synchronous hand more systematically than the asynchronous hand as the visual delay applied to the asynchronous hand increased and synchrony therefore became easier to detect.

The current study makes different predictions for ASD performance depending on whether there is an over-reliance on proprioceptive processing or temporally extended visuo-tactile binding in ASD, thereby allowing us to directly test the evidence for both theories:

1. Over-reliance on proprioceptive inputs: If the participants in the ASD group rely more heavily upon proprioception, and weight this input more than other sensory information [8, 19], then they should reliably select the synchronous hand when it is also the veridical hand (i.e. in congruent conditions). In incongruent conditions, even at larger delay lengths (when the synchronous hand is more easily detectable), synchrony should not completely override conflicting proprioceptive inputs. Consequently, they should not consistently embody the synchronous hand. This is in contrast to TD controls who should predominantly choose the synchronous hand due to the overriding saliency of synchrony, especially at longer delays.
2. Atypical visuo-tactile temporal binding: If children with ASD show temporally extended sensory binding [14, 16], then a longer than normal time period can elapse between multiple sensory inputs and integration of these still takes place. The TD controls should detect and chose the synchronous hand more consistently as the visuo-tactile delay applied to the asynchronous hand increases while one of two potential patterns of behaviour could be seen in the ASD group. The first is that there will be no effect of delay length (i.e. the synchronous hand will not be chosen more frequently at longer versus shorter delay lengths) if a delay length of more than 300 ms is needed before synchronous and asynchronous visuo-tactile inputs can be reliably distinguished. The second is that the delay length at which the ASD group is able to consistently discriminate and embody the synchronous hand should be longer than that seen in TD controls.

## Results and discussion

### Data analysis

In order to test the evidence for two opposing accounts of atypical sensory integration in ASD, we were interested in the extent to which the ASD group chose the synchronous hand across different conditions and in comparison to TD controls. There were two trials in each condition; therefore, each participant could choose the synchronous hand once, twice or not at all in each condition. Chi-square analyses were conducted for each group for each condition to assess whether the number of participants choosing the synchronous hand was more than expected if the group were performing at chance level, i.e. not performing systematically (Table 2). Bonferroni corrections were used such that all analyses comparing results against chance are reported at a .0003 level of significance.

**Table 2:** Chi-square analyses comparing the frequency of individuals choosing the synchronous hand against chance level in each group

	Congruent 60 ms	Congruent 180 ms	Congruent 300 ms	Incongruent 60 ms	Incongruent 180 ms	Incongruent 300 ms
CA	*χ*2(2) = 46.45, *p* < .001*	*χ*2(2) = 51.69, *p* < .001*	*χ*2(2) = 71.83, *p* < .001*	*χ*2(2) = 1.75, *p* = .42	*χ*2(2) = 35.14, *p* < .001*	*χ*2(2) = 29.15, *p* < .001*
MA	*χ*2(2) = 11.69, *p* = .003*	*χ*2(2) = 19.35, *p* < .001*	*χ*2(2) = 30.72, *p* < .001*	*χ*2(2) = 5.28, *p* = .07	*χ*2(2) = 3.41, *p* = .18	*χ*2(2) = 14.45, *p* < .001*
ASD	*χ*2(2) = 1.14, *p* = .57	*χ*2(2) = 14.45, *p* < .001*	*χ*2(2) = 11.62, *p* = .003*	*χ*2(2) = 2.31, *p* = .32	*χ*2(2) = 2.52, *p* = .28	*χ*2(2) = 6.93 *p* = .03

*CA* chronological-age-matched group, *MA* verbal mental-age-matched group, *ASD* autism spectrum disorder group

*Indicates performance that is significantly different to chance at .0003 level of significance

Chi-square analyses were also conducted to assess whether there were significant group differences in the frequency that participants chose the synchronous hand (Table 3). Although some of these chi-square group comparisons had more than 20 % of cases with expected frequencies less than five, it has been demonstrated that, when this occurs, it is extremely unlikely that an increase in type 1 errors will occur [35]. Nonetheless, significance levels were set at .025 to protect against this.

**Table 3:** Between-group chi-square analyses comparing the number of participants choosing the synchronous hand in the chronological age (CA)-matched group versus the autism spectrum disorder (ASD) group and the verbal mental age (MA)-matched group versus the ASD group

	Congruent 60 ms	Congruent 180 ms	Congruent 300 ms	Incongruent 60 ms	Incongruent 180 ms	Incongruent 300 ms
CA vs. ASD	*χ*2(2) = 18.79	*χ*2(2) = 5.17	*χ*2(2) = 12.66	*χ*2(2) = .73	*χ*2(2) = 10.27	*χ*2(2) = 12
	*p* < .001*	*p* = .075	*p* = .002*	*p* = .69	*p* = .006*	*p* = .002*
MA vs. ASD	*χ*2(2) = 5.29	*χ*2(2) = 1.08	*χ*2(2) = 2.19	*χ*2(2) = 1.23	*χ*2(2) = .08	*χ*2(2) = 1.72
	*p* = .07	*p* = .58	*p* = .34	*p* = .54	*p* = .96	*p* = .41

*Indicates significant group difference at .025 level of significance

### Results

**Fig. 3:**
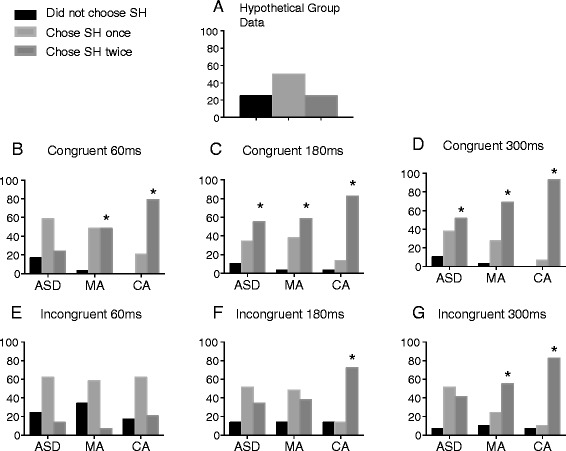
Chi-square results. *Y* axis = number of participants. **a** Hypothetical data showing a group choosing the synchronous hand at chance level. **b**–**g** Chi-square analyses comparing the frequency of individuals choosing the SH against chance level. *Asterisks* indicate performance that is significantly different to chance at .003 level of significance

Chance level per condition equates to 25 % of the group not choosing the synchronous hand, 50 % choosing the synchronous hand in one trial and 25 % choosing it in both trials. For comparison purposes, Fig. 3a shows what the frequency data would look like if a group’s performance was at chance level. Figure 3b–g displays the frequency that participants chose the synchronous hand in each group in each condition. These show that, across conditions, both TD groups chose the synchronous hand more than the ASD group. Across groups, the synchronous hand was chosen more in congruent, compared to incongruent, conditions and at longer, compared to shorter, delay lengths.

**Fig. 4:**
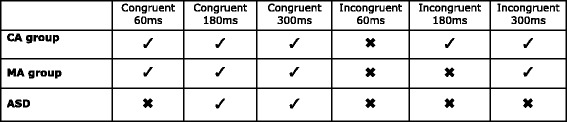
Results. *Ticks* = chose synchronous hand significantly above chance. *Crosses* = did not choose the synchronous hand significantly above chance. Results of the chi square results for comparison with Fig. 2. (predictions)

As predicted (see Fig. 2), both TD groups chose the synchronous hand above chance level in all congruent conditions. Children with ASD, though, did not consistently choose the synchronous hand in the congruent 60-ms condition but did so in the congruent 180- and 300-ms delay conditions, signifying that a 60-ms delay length was difficult for ASD children to detect. Between groups, chi-square analyses comparing the frequency for choosing the synchronous hand are shown in Table 3. These found no significant differences between the ASD group and the MA group, while the CA group chose the synchronous hand significantly more often than the ASD group in the congruent 60-ms (*χ*2 (2) = 18.79 *p* < . 001) and 300-ms conditions (*χ*2 (2) = 12.66 *p* = . 002). If there had been a fundamental over-reliance on proprioception, then the synchronous hand should not have been chosen in any incongruent conditions but should have been selected in all congruent conditions, even when the delay was short, yet this pattern of data was clearly not observed (see Fig. 4). Without detecting and distinguishing synchronous from asynchronous inputs in the 60-ms condition, proprioceptive information alone was not sufficient for the ASD group to embody the (veridical) synchronous hand. Thus, these results provide direct evidence against the idea that there is a fundamental bias for processing proprioceptive information over other sensory inputs. With an increased delay length, however, the combined weighting of visual, tactile and proprioceptive inputs led to embodiment of the synchronous hand in congruent 180- and 300-ms conditions. Therefore, compared to age-matched controls, the ASD group appear to need a longer delay between synchronous and asynchronous inputs before they can clearly discern the synchronous hand, indicating extended and less precise sensory binding. Though previous research has demonstrated this for auditory-visual processing in ASD [14], this is the first study to provide definitive evidence for temporally extended visuo-tactile binding in this population.

The CA group chose the synchronous hand above chance level in the incongruent 180- and 300-ms conditions while the MA group only did so in the 300-ms condition (see Fig. 4). These results indicate that the TD children were guided by visuo-tactile temporal synchrony, even when this information was incongruent with proprioceptive information. This tendency is seen in RHI studies with both children [32] and adults [20] and supernumerary limb illusions [29–31, 36]. Although the synchronous hand was chosen less in incongruent versus congruent conditions for TD controls, our findings are consistent with the broader embodiment literature in that we are more likely to embody a fake hand when there is less proprioceptive discrepancy between it and our real unseen hand [37–39]. This is also in keeping with data from Paton et al.’s RHI study [19] in which the illusion was stronger for TD individuals in conditions when video goggles were worn such that there was no proprioceptive discrepancy between the rubber and the real hand.

Chance level performance by the control groups in the incongruent 60-ms condition suggests that the synchronous hand was difficult to detect and not sufficient to completely override conflicting proprioceptive inputs. Though the MA group required an even longer delay (300 versus 180 ms) to reliably choose the synchronous hand in incongruent conditions than the CA group, this is most likely due to age-related differences in sensory binding: younger children show temporally extended binding for audio-visual stimuli [40] and are less sensitive to violations of visual-proprioceptive synchrony [41]. The older CA group would likely have been more sensitive to the discrepancy between synchronous and asynchronous visuo-tactile information in the 180-ms delay conditions than the younger MA group, which, consequently, did not systematically embody the synchronous hand in that condition. These observations are further strengthened by between group analyses, which revealed that the CA group chose the synchronous hand significantly more often than the ASD group in the incongruent 180-ms (*χ*2 (2) = 10.27 *p* = . 006) and 300-ms conditions (*χ*2 (2) = 12, *p* = . 002), but there were no differences between the ASD and MA group. Thus, the ASD group was performing significantly differently to CA-matched TD children but was in line with younger TD children, demonstrating a developmental delay in visuo-tactile temporal binding.

The non-significant difference in performance between the ASD and CA group in the congruent 180-ms condition is likely an artefact related to different rates of improvement between the groups, since the trend for more children in the CA group to choose the synchronous hand is still present in this condition. In the congruent 60-ms condition, detecting the delay is very difficult for the ASD group, while in the congruent 300-ms condition, it is very easy for the CA group. Thus, group differences are exaggerated at these two extremes. In the congruent 180-ms condition, the ASD group is able to perform above chance in selecting the synchronous hand with congruent proprioceptive information; therefore, the difference in performance between the CA and ASD groups is not as strong at this point.

ASD performance in the congruent conditions points to temporally extended visuo-tactile binding relative to controls (compare Fig. 2 (predictions) with Fig. 4 (results)). If this was the only cause of atypical sensory processing, though, then once the synchronous hand can be clearly detected, the ASD group should also choose the synchronous hand above chance in incongruent conditions. In contrast to the TD controls, however, they did not choose the synchronous hand significantly above chance in any incongruent condition. Moreover, they did not switch to systematically choosing the veridical hand in incongruent conditions as predicted by a fundamental over-reliance on proprioception. Instead, our findings can be interpreted within a framework of reduced detection of, or sensitivity towards, amodal information in ASD [6]. This refers to inputs that are not specific to one particular sensory modality but can instead be present across multiple senses, such as temporal synchrony. Previous research [6, 42] has established that, in typical development, amodal information is selectively attended to and processed before modal specific inputs (e.g. proprioception) and is used to distinguish the ‘self’ from ‘other’ [41, 43]. Our findings are certainly in line with this theory: TD children seem sensitive to synchrony between the seen and felt touch and automatically bind sensory events together on that basis. They may use this to guide their attention towards, and embody, the synchronous hand, even when this information is incongruent with proprioceptive inputs. Additionally, amodal binding may allow them to distinguish between relevant and unrelated sensory information. Thus, TD children may have used the discrepancy between synchronous and asynchronous information to determine not only their actual hand but also which hand was not theirs.

In future research, it would be useful to include an additional control condition in which both hands were synchronous or both hands were asynchronous as this would help to fully disentangle the effect of synchronicity and the effect of proprioceptive congruency.

Nonetheless, overall, our findings provide clear evidence that children with ASD are not guided by the amodal properties of stimuli in the same way as TD children. In contrast to the TD controls, at delays of 300 ms, the ASD group was only sensitive to visuo-tactile synchrony when this information was congruent with modality-specific proprioceptive information. This indicates that the synchrony of seen and felt touch does not ‘pop out’ as meaningful or hold a special significance for children with ASD as it does in typical development. Consequently, visuo-tactile and proprioceptive information seems to be weighted such that neither the synchronous hand nor the veridical hand is consistently chosen in any incongruent conditions. Hence, it is not that there is an over-reliance on proprioception across all contexts in ASD, rather, unlike the TD controls, synchrony does not override proprioception when the two are incongruent, suggesting the inputs may be more equally weighted.

Although there is a growing body of research on visual-auditory processing in ASD (e.g. [14-16, 44] the current findings fill a notable gap in this literature by furthering our understanding of the processes underlying atypical visual, tactile and proprioceptive integration. It has been suggested that an extended sensory temporal binding window could underlie reduced sensitivity to amodal information [6] since distinctions between synchronous and asynchronous inputs may not be made if sensory binding is more extended and imprecise. Our findings lend important weight to this proposal—for the first time, it is possible to confidently implicate temporally extended visuo-tactile binding in the ASD behavioural profile, alongside reduced sensitivity to (and, thus, reliance on) amodal information. Atypical temporal binding would increase the likelihood of information from unrelated events being inappropriately bound together, which could underlie the sensory sensitivities in ASD [18]. Furthermore, Stevenson et al. [44] demonstrated a relationship between low-level audio-visual temporal binding deficits and poor speech processing abilities in ASD. It is likely that temporally extended visuo-tactile binding could also have cascading effects on higher order processes, including social functioning. Indeed, both this and reduced sensitivity to amodal information would impair the visuo-tactile integration necessary for acquiring a sense of ‘self’ [43] which underlies the social processes that are compromised in ASD, such as empathy and imitation.

Moreover, research supports a genetic influence on sensory sensitivities in the general population [45] and in ASD specifically [46]. The current findings help to identify specific mechanisms that could underlie these sensory atypicalities. Future research should investigate whether temporally extended sensory binding and/or reduced detection of amodal information is present in the siblings of children with ASD, which would further elucidate the genetic contribution to sensory sensitivities in ASD.

## Conclusions

Beyond understanding a prevalent developmental condition, these results have important implications for evidence-based interventions in ASD. Future research could employ psychophysical methods to determine each individual’s temporal binding window, which would be valuable for measuring the effectiveness of interventions. Since the visuo-auditory binding window can be narrowed using multisensory perceptual feedback training [47, 48], it is likely that the visuo-tactile binding window is similarly malleable. A narrower window would increase the ease with which amodal information could be detected and distinguished from modal specific inputs, which in turn could alleviate the sensory sensitivities, reduced body ownership and social impairments characterising ASD. This may therefore provide an efficient and tractable form of intervention that can alleviate difficulties across the behavioural profile.

## Abbreviations

ASD: autism spectrum disorders
CA: chronological age-matched controls
DSM-5: Diagnostic and Statistical Manual of Mental Disorders (fifth edition)
MA: verbal mental-age-matched controls
MSI: multisensory integration
RHI: rubber hand illusion
SAS: Social Aptitudes Scale
SCQ: Social Communication Questionnaire
SWAN: Strengths and Weaknesses of ADHD Symptoms and Normal Behavior Scale
TD: typically developing
